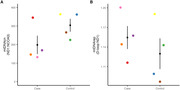# Developing an optimized protocol to measure mitochondrial phenotypes as a blood‐based biomarker for mitochondrial dysfunction in AD

**DOI:** 10.1002/alz.095352

**Published:** 2025-01-09

**Authors:** Aadrita Chatterjee, Daniel W. Sirkis, Taylor Johnson, Thu Pham, Bruce L. Miller, Gil D. Rabinovici, Heather M Wilkins, Kristine Yaffe, Russell H Swerdlow, Jennifer S. Yokoyama, Shea J Andrews

**Affiliations:** ^1^ UCSF, San Francisco, CA USA; ^2^ Memory and Aging Center, UCSF Weill Institute for Neurosciences, San Francisco, CA USA; ^3^ Memory and Aging Center, UCSF Weill Institute for Neurosciences, University of California, San Francisco, San Francisco, CA USA; ^4^ Weill Institute for Neurosciences, University of California, San Francisco, San Francisco, CA USA; ^5^ University of Kansas Medical Center, Kansas City, KS USA; ^6^ University of California San Francisco / San Francisco VA Medical Center, San Francisco, CA USA; ^7^ University of California, San Francisco, San Francisco, CA USA

## Abstract

**Background:**

Mitochondrial DNA copy number (mtDNAcn) quantifies the number of mitochondria genomes per nucleated cell, with reduced mtDNAcn being associated with increased Alzheimer’s disease (AD) neuropathology. Blood‐based mtDNAcn has technical confounders, such as DNA purification, and biological confounders, such as compensatory upregulation of mtDNA. Therefore, we optimized a protocol for mtDNAcn quantification using droplet digital PCR (ddPCR) by testing (i) whole peripheral blood mononuclear cells (PBMCs) vs platelet‐depleted PBMCs, (ii) column‐based DNA extraction vs cell lysate, and (iii) mitochondrial DNA replication (mtDNArep). We found that whole PBMCs overestimated mtDNAcn levels, and column‐based DNA extraction resulted in a partial mtDNA loss; therefore, platelet‐depleted PBMC lysates yielded the most accurate measurement. Here, we applied this optimized protocol to quantify mtDNAcn in early‐onset AD (EOAD) cases and controls.

**Method:**

Platelets were depleted from whole PBMCs using a magnetic‐activated cell sorter against the platelet marker CD61. Platelet‐depleted PBMCs were lysed and DNA concentration was measured using Qubit fluorometric quantification. We used platelet‐depleted PBMC lysates from four female EOAD cases (aged 60.6 ± 1.4 years) and four female controls (aged 59 ± 2.5 years). mtDNAcn was assessed using ddPCR with multiplexed wells for mitochondrial signals (using D‐Loop and ND1) at a 0.05 ng DNA input amount and non‐multiplexed wells for the nuclear signal (using NCOA3) at a 0.25 ng DNA input amount. We evaluated the association of EOAD status with mtDNAcn ((ND1/NCOA3)*2) and mtDNArep (D‐loop/ND1) using linear regressions adjusting for age.

**Result:**

We found that mtDNAcn was lower in EOAD compared to controls, and our results were marginally significant (b [SE] = 127 [51.9], p = 0.058; Figure 1A). However, we observed no significant difference in mtDNArep between cases and controls (b [SE] = ‐0.019 [0.031], p = 0.56; Figure 1B).

**Conclusion:**

Consistent with prior literature, we found that mtDNAcn is lower in EOAD cases. These results demonstrate the feasibility of our protocol for quantifying mtDNAcn and mtDNArep to use as fluid biomarkers for mitochondrial dysfunction. Future work will adjust for blood cell type abundance and evaluate the association of mtDNAcn and mtDNArep with AD in larger, diverse cohorts to determine whether mitochondrial dysfunction causes, mediates, or is a by‐product of AD pathogenesis.